# Supplementary material for: Environmental modulation of global epistasis in a drug resistance fitness landscape
Source: Nat Commun. 2023 Dec 5;14:8055. doi: 10.1038/s41467-023-43806-x (PMC10698197; doi:10.1038/s41467-023-43806-x)
Supplement: Supplementary file 1 — Suppplementary Information [file 41467_2023_43806_MOESM1_ESM.pdf]

Supplementary Information for

Environmental modulation of global epistasis in a drug resistance fitness landscape

Juan Diaz-Colunga, Alvaro Sanchez, C. Brandon Ogbunugafor

Correspondence:
JDC: juan.diazcolunga@yale.edu, AS: alvaro.sanchez@usal.es, CBO: brandon.ogbunu@yale.edu

Supplementary Notes

Derivation of equations 1-5

We consider a genome of L loci, such that a given genotype can be represented by a vector $\mathbf{x} = (x_1, x_2, \cdots, x_L)$. The element x_i of the vector is +1 or −1 depending on whether locus i is or is not mutated, respectively. The fitness $f(\mathbf{x})$ of such a genotype can be generically expressed as

$$f(\mathbf{x}) = \bar{f} + \sum_i \delta_i x_i + \sum_i \sum_{j>i} \delta_{ij} x_i x_j + \sum_i \sum_{j>i} \sum_{k>j>i} \delta_{ijk} x_i x_j x_k + \cdots \tag{S1}$$

where δ_i, δ_{ij}, δ_{ijk}, etc. are parameters capturing the interactions between loci at different orders, and $\bar{f}$ is the average fitness across all genotypes. We denote B a genotype not carrying mutation i, that is, one potential genetic background for mutation i. Recent work has shown that the variance in fitness across such backgrounds is given by[48]

$$\operatorname{var} f(B) = \sum_{j\neq i} \left(\delta_j - \delta_{ij}\right)^2 + \sum_{k\neq i} \sum_{j>k} \left(\delta_{jk} - \delta_{ijk}\right)^2 + \cdots \tag{S2}$$

and the variance in the fitness effect of mutation i across backgrounds is

$$\operatorname{var} \Delta f_i = 4 \sum_{j\neq i} \delta_{ij}^2 + 4 \sum_{k\neq i} \sum_{j>k} \delta_{ijk}^2 + \cdots \tag{S3}$$

In turn, the covariance between the fitness effect Δf_i and the fitness of the background is

$$\operatorname{cov}\Big(\Delta f_i, f(B)\Big) = 2 \sum_{j\neq i} \left(\delta_j - \delta_{ij}\right) \delta_{ij} + 2 \sum_{k\neq i} \sum_{j>k} \left(\delta_{jk} - \delta_{ijk}\right) \delta_{ijk} + \cdots \tag{S4}$$

It can be shown[49] that the average fitness effect of mutation j (see Box 1 in main text) is just

$$\langle \Delta f_j \rangle = 2\delta_j \tag{S5}$$

and the average magnitude of epistasis between mutations i and j (ϵ_{ij} as defined in Box 1) is

$$\langle \epsilon_{ij} \rangle = 4\delta_{ij} \tag{S6}$$

Introducing equations S5 and S6 into equations S2-S4, and keeping only the lowest-order terms[49] yields the following approximation for the variance ratio:

$$\frac{\operatorname{var} \Delta f_i}{\operatorname{var} f(B)} \approx \frac{\sum_{j\neq i} \langle \epsilon_{ij} \rangle^2}{\sum_{j\neq i} \langle \Delta f_j \rangle^2} = \sum_{j\neq i} \frac{\langle \epsilon_{ij} \rangle^2}{\langle \Delta f_j \rangle^2} \frac{\langle \Delta f_j \rangle^2}{\sum_{k\neq i} \langle \Delta f_k \rangle^2} = \sum_{j\neq i} \omega_{ij} \beta_{ij}^2 \tag{S7}$$

where β_{ij} and ω_{ij} are defined in Box 1. The coefficient of determination of the regression between Δf_i and $f(B)$ (R_i^2 for mutation i), which we use as a quantification of the degree of global epistasis for that mutation (see main text), can be similarly approximated as

$$R_i^2 = \frac{\operatorname{cov}^2\Big(\Delta f_i, f(B)\Big)}{\operatorname{var} \Delta f_i \cdot \operatorname{var} f(B)} \approx \frac{\left(\sum_{j\neq i} \langle \epsilon_{ij} \rangle \langle \Delta f_j \rangle\right)^2}{\sum_{j\neq i} \langle \epsilon_{ij} \rangle^2 \ \sum_{j\neq i} \langle \Delta f_j \rangle^2} = \frac{\left(\sum_{j\neq i} \frac{\langle \epsilon_{ij} \rangle}{\langle \Delta f_j \rangle} \frac{\langle \Delta f_j \rangle^2}{\sum_{k\neq i} \langle \Delta f_k \rangle^2}\right)^2}{\sum_{j\neq i} \frac{\langle \epsilon_{ij} \rangle^2}{\langle \Delta f_j \rangle^2} \frac{\langle \Delta f_j \rangle^2}{\sum_{k\neq i} \langle \Delta f_k \rangle^2}} = \frac{\left(\sum_{j\neq i} \omega_{ij} \beta_{ij}\right)^2}{\sum_{j\neq i} \omega_{ij} \beta_{ij}^2} \tag{S8}$$

Finally, the slope of the regression between Δf_i and $f(B)$ (denoted b_i) can be approximated as

$$b_i = \frac{\operatorname{cov}\Big(\Delta f_i, f(B)\Big)}{\operatorname{var} f(B)} \approx \frac{\sum_{j\neq i} \langle \epsilon_{ij} \rangle \langle \Delta f_j \rangle}{\sum_{j\neq i} \langle \Delta f_j \rangle^2} = \sum_{j\neq i} \frac{\langle \epsilon_{ij} \rangle}{\langle \Delta f_j \rangle} \frac{\langle \Delta f_j \rangle^2}{\sum_{k\neq i} \langle \Delta f_k \rangle^2} = \sum_{j\neq i} \omega_{ij} \beta_{ij} \tag{S9}$$

Empirical global epistasis patterns are not a consequence of regression to the mean

Correlations between the fitness effect of a mutation and the fitness of its genetic background can emerge as a simple consequence of regression to the mean. Intuitively, the fitness effect of a mutation will necessarily be negative if the genetic background corresponds to the highest-fitness genotype, and positive if it corresponds to the lowest-fitness genotype. Statistically, the fitness effect of a mutation can thus be expected to be either negative or, at best, positive but small, when the background fitness is high and vice-versa. This may lead to negative correlations between Δf and $f(B)$. Importantly, a similar argument can be made regarding measurement error: correlations in the errors for $f(B)$ and Δf can similarly lead to spurious negative global epistasis slopes[53].

To test the extent to which this effect may drive the global epistasis patterns observed in our landscape, we randomized the pairing between genotypes and fitness values in our dataset. Such a randomized landscape has no "global" structure, and thus the correlations we might observe between $f(B)$ and Δf may be entirely attributed to statistical regression to the mean. We generated 500 randomizations for each drug dose, and each time we quantified the strength of epistasis (variance ratio $\mathrm{var}\,\Delta f\,/\,\mathrm{var}\,f(B)$) and the degree to which epistasis is "global" (R^2 of the linear regression between $f(B)$ and Δf) for each mutation as explained in the main text. As expected, we often found negative relationships between $f(B)$ and Δf, leading to non-zero values for the R^2. Importantly, however, in fig. 6 we show that the empirically observed patterns of global epistasis in our landscape are largely not compatible with the $f(B)$-vs-Δf regressions found in the randomization controls.

Supplementary Figures

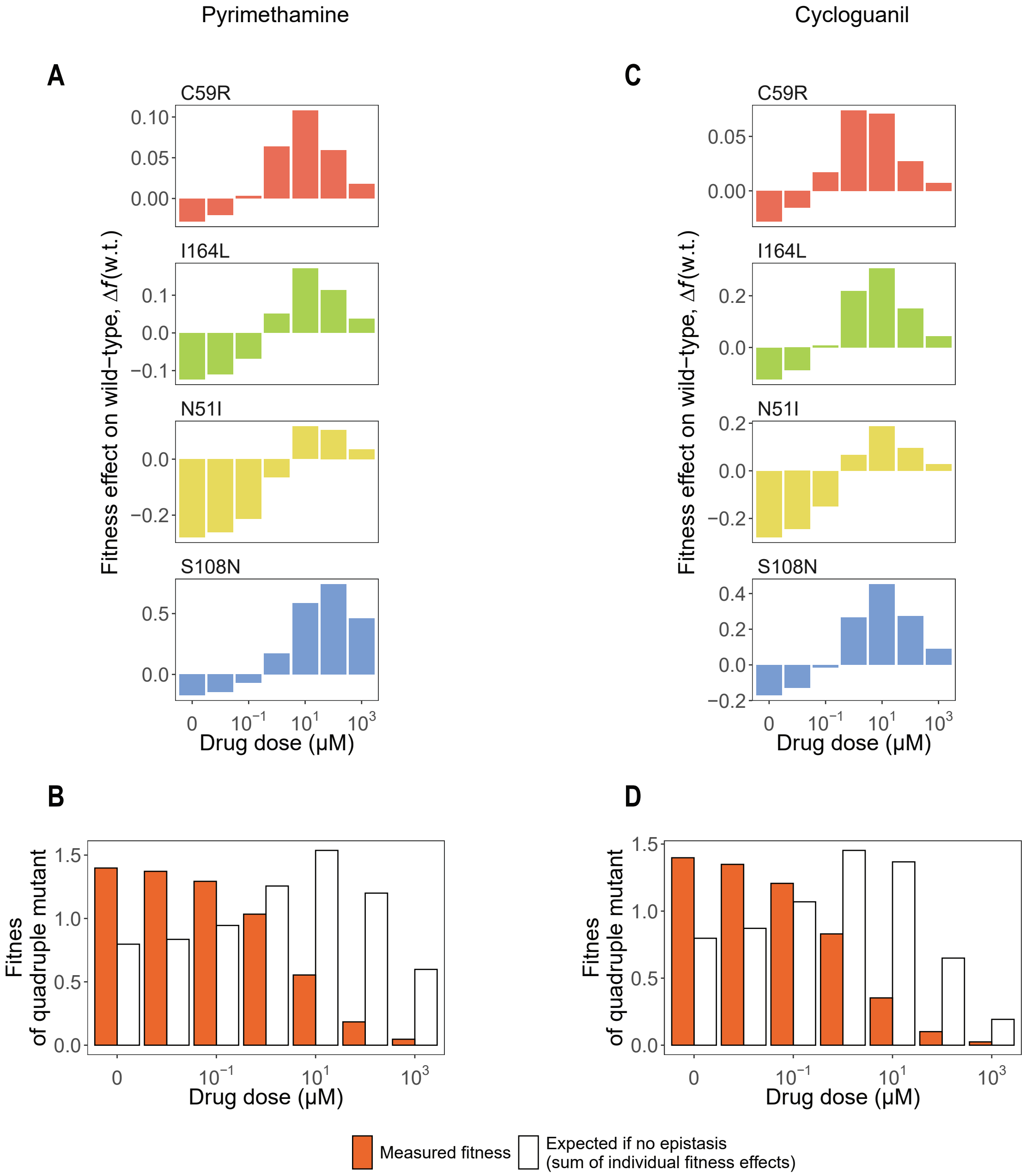

Supplementary Figure 1. Mutational fitness effects on wild-type genotype and fitness of the quadruple mutant across drug doses. (**A**) Fitness effects of the four mutations on the wild-type genotype across pyrimethamine doses. All mutations are individually deleterious at low doses ($\Delta f < 0$) and beneficial at high doses ($\Delta f > 0$). (**B**) In the absence of epistasis, all four fitness effects would combine additively in the quadruple mutant: hollow bars represent $f(\text{w.t.}) + \sum_i \Delta f_i(\text{w.t.})$, where $f(\text{w.t.})$ is the fitness of the wild-type genotype and $\Delta f_i(\text{w.t.})$ is the fitness effect of mutation i on the wild-type genotype. In reality, epistasis makes it so the empirical fitness of the quadruple mutant (orange bars) is larger than this additive expectation at low doses, and smaller at high doses: even if epistasis decreases the fitness of the quadruple mutant in the presence of the drug, it also palliates the fitness cost of the four mutations when there is no drug in the environment. (**C**-**D**) Same as two last panels for cycloguanil.

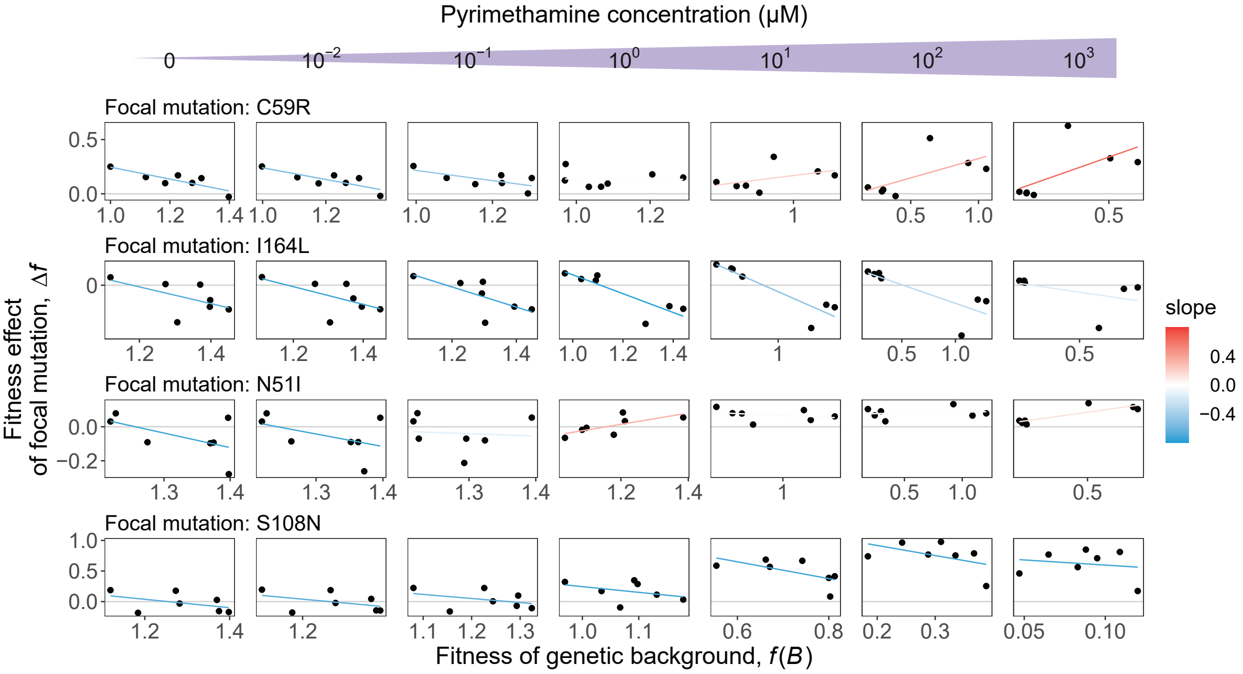

Supplementary Figure 2. Environmental modulation of global epistasis patterns in a pyrimethamine concentration gradient. We represent the global epistasis patterns for all four mutations (rows) in every dose of pyrimethamine from 0 to 10^3 μM (columns). Note that the first row in this figure corresponds to Fig. 1D of the main text and is included here for completeness.

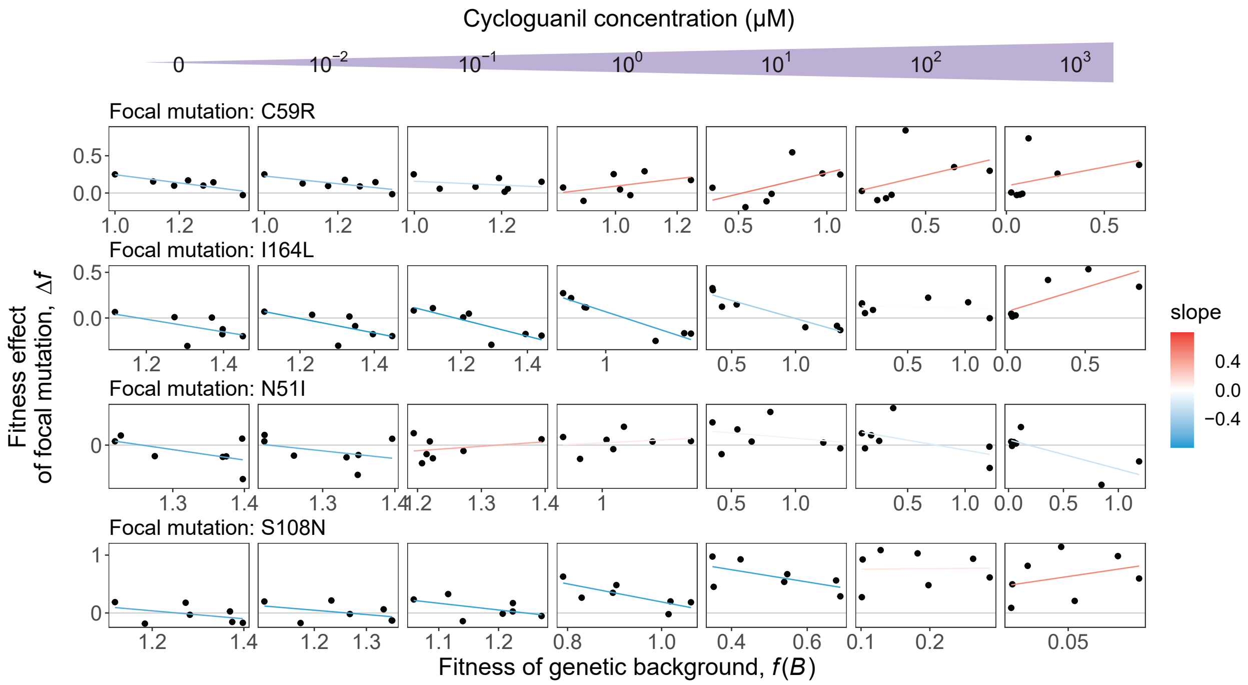

Supplementary Figure 3. Environmental modulation of global epistasis patterns in a cycloguanil concentration gradient. Global epistasis patterns for all four mutations (rows) in every dose of cycloguanil from 0 to 10^3 µM (columns).

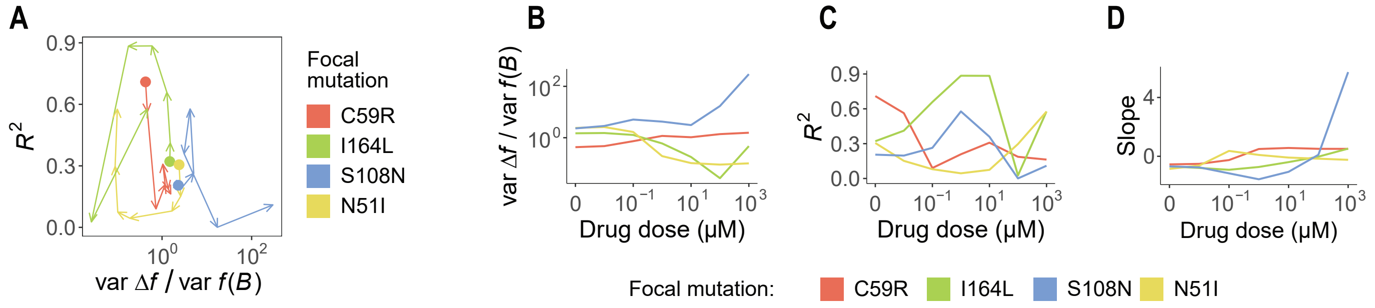

Supplementary Figure 4. Environmental modulation of global epistasis across a cycloguanil concentration gradient. (**A**) Trajectories followed by each of the four mutations in the "epistasis map" as cycloguanil dose increases from 0 to 10^3 μM. (**B**-**D**) The variance ratio, R^2, and slope for each mutation are modulated by drug dose.

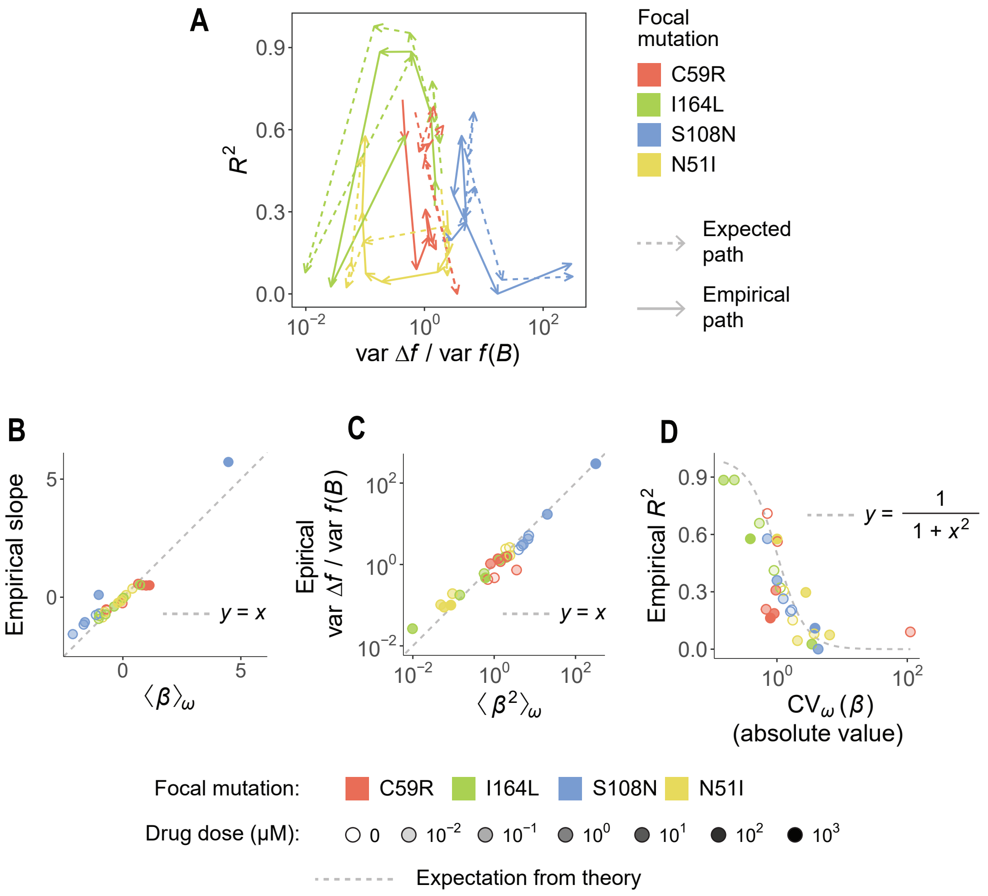

Supplementary Figure 5. The moments of the distribution of effective interactions estimate the strength and shape of global epistasis across a cycloguanil gradient. We reproduce the analyses shown in Fig. 4 of the main text, here using the cycloguanil data. (**A**) We show the paths that mutations follow in the "map of epistasis" as drug concentration increases. Solid lines: paths obtained from empirically quantifying the variance ratios and R^2. Dashed lines: paths obtained by estimating variance ratios and R^2 from equations 4 and 5 in Box 1. (**B**-**D**) We compare the empirically obtained values for the variance ratio, R^2, and global epistasis slope for every mutation in the dataset and at all concentrations of cycloguanil with the values estimated from each mutation's distribution of effective interactions (equations 1, 4, and 5 in Box 1).

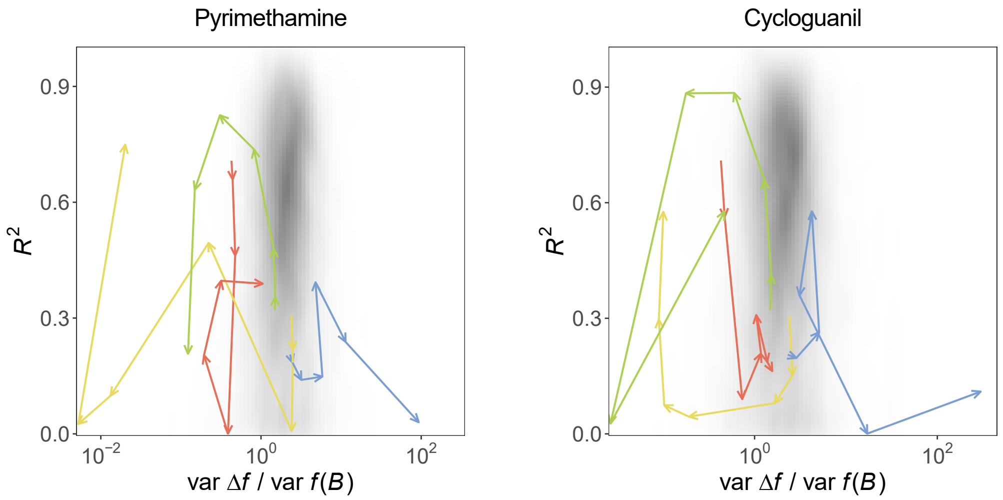

Supplementary Figure 6. Empirical global epistasis patterns in our landscape are not a consequence of regression to the mean. We randomized the mapping between genomes and fitness in our dataset. We generated 500 randomizations for each drug dose. We quantified the variance ratio and R^2 of the regression between $f(B)$ and Δf for all mutations in each of these randomized landscapes. Black shaded regions represent regions of the "epistasis map" that were most commonly occupied by mutations in the randomized landscapes. The empirically observed patterns of global epistasis in our true landscape (colored lines) are generally not compatible with the randomization controls.
