# Supplementary material for: Environmental modulation of global epistasis in a drug resistance fitness landscape
Source: Nat Commun. 2023 Dec 5;14:8055. doi: 10.1038/s41467-023-43806-x (PMC10698197; doi:10.1038/s41467-023-43806-x)
Supplement: Supplementary file 3 — Reporting Summary [file 41467_2023_43806_MOESM3_ESM.pdf]

nature portfolio

Corresponding author(s): Juan Diaz-Colunga, Alvaro Sanchez, C. Brandon Ogbunugafor

Last updated by author(s): Nov 3, 2023

Reporting Summary

Nature Portfolio wishes to improve the reproducibility of the work that we publish. This form provides structure for consistency and transparency in reporting. For further information on Nature Portfolio policies, see our Editorial Policies and the Editorial Policy Checklist.

Statistics

For all statistical analyses, confirm that the following items are present in the figure legend, table legend, main text, or Methods section.

n/a	Confirmed	
☐	☒	The exact sample size (*n*) for each experimental group/condition, given as a discrete number and unit of measurement
☒	☐	A statement on whether measurements were taken from distinct samples or whether the same sample was measured repeatedly
☒	☐	The statistical test(s) used AND whether they are one- or two-sided<br>*Only common tests should be described solely by name; describe more complex techniques in the Methods section.*
☒	☐	A description of all covariates tested
☒	☐	A description of any assumptions or corrections, such as tests of normality and adjustment for multiple comparisons
☐	☒	A full description of the statistical parameters including central tendency (e.g. means) or other basic estimates (e.g. regression coefficient) AND variation (e.g. standard deviation) or associated estimates of uncertainty (e.g. confidence intervals)
☒	☐	For null hypothesis testing, the test statistic (e.g. *F*, *t*, *r*) with confidence intervals, effect sizes, degrees of freedom and *P* value noted<br>*Give P values as exact values whenever suitable.*
☒	☐	For Bayesian analysis, information on the choice of priors and Markov chain Monte Carlo settings
☒	☐	For hierarchical and complex designs, identification of the appropriate level for tests and full reporting of outcomes
☒	☐	Estimates of effect sizes (e.g. Cohen's *d*, Pearson's *r*), indicating how they were calculated

Our web collection on statistics for biologists contains articles on many of the points above.

Software and code

Policy information about availability of computer code

Data collection	No software was used for data collection.
Data analysis	Analyses were performed using R version 4.1.2. All code necessary to reproduce the analyses is available at https://github.com/jdiazc9/env_global_epist (DOI: doi.org/10.5281/zenodo.10067163).

For manuscripts utilizing custom algorithms or software that are central to the research but not yet described in published literature, software must be made available to editors and reviewers. We strongly encourage code deposition in a community repository (e.g. GitHub). See the Nature Portfolio guidelines for submitting code & software for further information.

Data

Policy information about availability of data

All manuscripts must include a data availability statement. This statement should provide the following information, where applicable:

- Accession codes, unique identifiers, or web links for publicly available datasets
- A description of any restrictions on data availability
- For clinical datasets or third party data, please ensure that the statement adheres to our policy

The data analyzed in this study were obtained from the original publications and is also available at https://github.com/jdiazc9/env_global_epist (DOI: doi.org/10.5281/zenodo.10067163).

Research involving human participants, their data, or biological material

Policy information about studies with human participants or human data. See also policy information about sex, gender (identity/presentation), and sexual orientation and race, ethnicity and racism.

Reporting on sex and gender	No human participants in this study.
Reporting on race, ethnicity, or other socially relevant groupings	n/a
Population characteristics	n/a
Recruitment	n/a
Ethics oversight	n/a

Note that full information on the approval of the study protocol must also be provided in the manuscript.

Field-specific reporting

Please select the one below that is the best fit for your research. If you are not sure, read the appropriate sections before making your selection.

☐ Life sciences ☐ Behavioural & social sciences ☒ Ecological, evolutionary & environmental sciences

For a reference copy of the document with all sections, see nature.com/documents/nr-reporting-summary-flat.pdf

Ecological, evolutionary & environmental sciences study design

All studies must disclose on these points even when the disclosure is negative.

Study description	We analyze a dataset consisting of fitness measurements for 15 genotypes of the P. falciparum parasite across a concentraation gradient of two aantiparasitic drugs. Fitness was quantified as the growth rate relative to that of the slowest-growing genotype in the absence of drug. We analyze the effect of each mutation on the fitness of the organism, considering mutation-by-mutation and mutation-by-environment (drug dose) interactions.
Research sample	Sample consists of a set of genotypes of the P. falciparum parasite, each carrying a different combination of four mutations. Out of the 16 potential combinations, one was disregarded for further analyses due to exhibiting incosistent fitness values in the original dataa source across two no-drug controls.
Sampling strategy	Due to the low dimensionality of the dataset, no sampling was considered. All genotypes but one (see "data exclusions" below) are part of the sample. All reported statistics were computed using the full sample.
Data collection	No new experimental data were collected for this study.
Timing and spatial scale	No new experimental data were collected for this study.
Data exclusions	Data for one genotype was excluded from the analysis due to inconsistent fitness values across two replicate controls. The final dataset contains 15 genotypes.
Reproducibility	No new experimental data were collected for this study. The source data consist of a single replicate per environment.
Randomization	The mapping between genotypes and fitness was not randomized as the aim was to quantify the fitness effect of each mutation on every specific background genotype.
Blinding	For the analyses in this study, blinding was not possible as the quantification of fitness effects requires prior knowledge on the identity of each genotype.

Did the study involve field work? ☐ Yes ☒ No

Reporting for specific materials, systems and methods

We require information from authors about some types of materials, experimental systems and methods used in many studies. Here, indicate whether each material, system or method listed is relevant to your study. If you are not sure if a list item applies to your research, read the appropriate section before selecting a response.

Materials & experimental systems

n/a	Involved in the study
☒	☐ Antibodies
☒	☐ Eukaryotic cell lines
☒	☐ Palaeontology and archaeology
☒	☐ Animals and other organisms
☒	☐ Clinical data
☒	☐ Dual use research of concern
☒	☐ Plants

Methods

n/a	Involved in the study
☒	☐ ChIP-seq
☒	☐ Flow cytometry
☒	☐ MRI-based neuroimaging
